
JOURNAL INFORMATION
==============================
NLM Title Abbreviation: ScientificWorldJournal
Journal ID: ScientificWorldJournal
NLM Journal ID: 101131163
Journal ID: TSWJ

The Scientific World Journal

ISSN: 2356-6140
EISSN: 1537-744X
Publisher: Wiley

ARTICLE INFORMATION
==============================
PMCID: PMC6083969
PMID: 30147611
DOI: 10.1100/tsw.2001.225
Article ID: 316782
Article version: 1
Subjects: Short Report

Regulation of Longevity by Insulin/Igf-1 Signaling, Sensory Neurons and the Germline in the Nematode C. Elegans

Kenyon Cynthia
Herbert Bayer Distinguished Professor of Biochemistry, University of California, 513 Parnassus Avenue, San Francisco, CA 94143-0448, USA
Publication date: 2001
Electronic publication date: 2001 Nov 6
Volume: 1
First page: 132
Last page: 132
Copyright: Copyright © 2001 Cynthia Kenyon.
Copyright year: 2001
License: This is an open access article distributed under the Creative Commons Attribution License, which permits unrestricted use, distribution, and reproduction in any medium, provided the original work is properly cited.
License URL: https://creativecommons.org/licenses/by/3.0/

==============================
Miami *Nature Biotechnology* Short Reports
*TheScientificWorld* (2001) 1 (S3), 132SR
ISSN 1532-2246; DOI 10.1100/tsw.2001.225

### REGULATION OF LONGEVITY BY INSULIN/IGF-1 SIGNALING, SENSORY NEURONS AND THE GERMLINE IN THE NEMATODE C. ELEGANS

Cynthia Kenyon

Herbert Bayer Distinguished Professor of Biochemistry, University of California, 513 Parnassus Avenue, San Francisco, CA 94143-0448
ckenyon@biochem.ucsf.edu

Aging is regulated hormonally in the nematode C. elegans. In my presentation, I will describe this elaborate endocrine system, which involves an insuln/IGF-1 signaling pathway, with inputs from the sensory tissues and the reproductive system. Our studies suggest that multiple hormones regulate the life span of this animal, including insulin/IGF-1-like hormones and a steroid hormone. Aging in this organism is extremely plastic; changes in any of a number of genes can extend youthfulness and double, or even quadruple, life span.
